# OUTPATIENT REHABILITATION CAN PREVENT SUICIDE AFTER ACQUIRED BRAIN INJURY

**DOI:** 10.2340/jrm.v58.45256

**Published:** 2026-07-21

**Authors:** Ann K.M. SÖRBO, Ragnhild I. BJÖRKLUND

**Affiliations:** 1 Institute of Neuroscience and Physiology, Department of Clinical Neuroscience, Sahlgrenska Academy, Gothenburg University, Gothenburg; 2 Clinic of Neurology, Rehabilitation and Specialized Care at Home, Södra Älvsborg Hospital, Borås; 3 Department of Research, Education and Innovation, Region Västra Götaland, Södra Älvsborg Hospital, Borås, Sweden

**Keywords:** brain injuries, suicide, rehabilitation

## Abstract

**Objective:**

The frequency of suicide after stroke and traumatic brain injury has been found to be higher than in the general population. This article presents an outpatient rehabilitation programme for adults with acquired brain injuries and compares the frequency of suicides among treated patients with that in a matched control group.

**Methods:**

A registry study was supplemented with reviews of medical records. Data were obtained from the Swedish Cause of Death Register, and 5 controls for each patient treated by the Outpatient Brain Injury Team were identified in the Swedish National Patient Register. Controls were matched by ICD-9 or ICD-10 diagnosis codes, age, gender, and date of first delivery of care.

**Results:**

A total of 591 patients were included in the treated group, and 2,932 individuals were included in the control group. No suicides were identified in the treated group, whereas 26 cases classified as intentional self-harm and a total of 40 suicides, including 14 probable suicides coded as events of undetermined intent, were identified among the controls.

**Conclusion:**

Treatment by a specialized outpatient rehabilitation team may help reduce suicide risk. A follow-up rehabilitation programme should be considered for patients who have sustained a brain injury.

## LAY ABSTRACT

The frequency of suicide after stroke and traumatic brain injury has been found to be higher than in the general population. This study compared patients from an outpatient rehabilitation programme for adults with acquired brain injuries with a matched control group. From the Swedish National Patient Register, 5 controls were selected for each treated patient, matched by diagnosis codes, age, gender, and date of first delivery of care. Data on causes of death were obtained from the Swedish Cause of Death Register for both groups. No suicides occurred among the treated group. In the control group, 26 individuals had cause of death recorded as “Intentional self-harm” and 14 as “Event of undetermined intent”. These findings suggest that treatment by a specialized outpatient rehabilitation team may be effective in preventing suicide, and that a structured follow-up rehabilitation programme should be offered to patients with brain injury.

The frequency of suicide after stroke, as well as after TBI, has been found to be higher than in the general population. Teasdale and Engberg published a Danish study in 2001 reporting a suicide risk of 83 per 100,000 individuals after stroke, compared with an expected 45 per 100,000 (1). The risk was highest during the first 5 years after stroke. In a study of 220,336 patients registered in Riksstroke (the Swedish Stroke Register), Eriksson et al. identified 1,217 suicide attempts, of which 260 were fatal (2). The frequency of fatal suicides was approximately twice as high per 100,000 person-years compared with a Swedish age-matched general population. Young age and male sex were associated with an increased risk of suicide attempt, with the highest risk occurring during the first 2 years after stroke.

Previous research has shown that depression is common after stroke, including aneurysmal subarachnoid haemorrhage (3–6), and after traumatic brain injury (TBI) including concussion (7, 8), and is 1 potential risk factor.

Fralick et al. presented a systematic review in 2016 (9) showing that mild traumatic brain injury (mTBI) increases the risk of suicide to 3 times that of the general population. A registry study by Madsen et al. in 2018 (10) reported an elevated risk of dying by suicide following TBI, particularly in cases of severe injury. The absolute risk was 41 per 100,000 person-years for individuals with TBI, compared with 20 per 100,000 person-years in the general population without a TBI diagnosis.

Studies have highlighted the need for research on supporting recovery (11) and reducing suicide rates in these patients (12). Evidence from other studies indicates that cognitive behavioural therapy (13), pharmacological treatment (14), and psychological interventions (15) targeting hopelessness are effective in treating depression and anxiety after stroke or TBI (16–19).

In 2020, Knight et al presented a qualitative study with the aim of exploring the experiences among individuals with TBI living with suicidality who pointed out the importance of long-term support (20).

Since 1998, the Department of Rehabilitation in Borås, Sweden, has operated a team providing outpatient interventions to adults with acquired brain injuries, known as the Outpatient Brain Injury Team (OBIT). As recovery and adaptation after a brain injury are often long-term processes, treatment may continue for several years. The team provides low-intensity treatment across different phases following the injury, as the timing of interventions is known to be important for rehabilitation effectiveness (21)**.** Pharmacological treatment and psychotherapy can be offered to address depression or severe crisis reactions.

From 1998 until 2020, to our clinical knowledge, no patient died by suicide during OBIT care. We aimed to determine whether any suicides occurred, including in the years following discharge from OBIT treatment, and to compare the frequency of suicides in the OBIT-treated group with a matched control group from the Swedish population.

As outpatient, long-term, structured specialist programmes for individuals with brain injuries are uncommon in Sweden, we expected a higher frequency of suicides in the control group compared with the OBIT group.

## MATERIALS AND METHODS

The programme began as a pilot project in 1995. The target group for OBIT care was, and remains, adult patients (approximately 18–65 years) with acquired brain injuries, encompassing a range of diagnoses and varying injury severity, corresponding to Glasgow Outcome Scale (GOSE) scores of 4–6 (22). This corresponds to Upper Severe Disability (cannot manage daily life independently for 24 h and requires help with planning and shopping), Lower Moderate Disability (can manage independently for 24 h but requires assistance, for instance to travel, and can work in sheltered employment), or Upper Moderate Disability (can work part-time and maintain a part-time social life). Patients with progressive neurological diseases were not accepted.

The patients could have pre-injury psychiatric diseases, alcohol and/or substance abuse, but no ongoing abuse during the treatment by the OBIT.

### The Outpatient Brain Injury Team group

The inclusion criteria for this study were that patients became ill between 1995 and 2015 and completed treatment between 1998 and 2020. All included patients (the OBIT group) had received at least 6 months of treatment from OBIT. The observation period was set to end in 2020, ensuring a minimum of 5 years of follow-up after illness onset for all included patients when the study commenced in 2021.

The OBIT team comprises a medical doctor, a neuropsychologist, a counsellor, an occupational therapist, and a medical secretary, with all members possessing some knowledge of neuropsychology. Patients requiring speech therapy or physiotherapy can receive treatment from consultants as needed. The team is led by a specialist in rehabilitation medicine. The programme begins with visits and assessments by all professionals. Patients complete various self-assessment forms prior to their first consultation, including the Montgomery–Åsberg Depression Rating Scale (MADRS-S) (23). All team members assess patients for clinical signs of depression.

The physician manages sick leave certificates and prescribes medication for depression and sleep disturbances, and for some patients also analgesics. There is collaboration with primary care, which oversees stroke prevention medication, hypertension, and other conditions or symptoms unrelated to the brain injury. Collaboration with other specialists, primarily neurologists and psychiatrists, is also arranged when needed.

The neuropsychologist conducts cognitive assessments and provides crisis counselling, as well as psychotherapy for depression, commonly based on cognitive behavioural therapy (CBT). Helping patients understand the consequences of their brain injury and why life has become more challenging is an important part of their crisis processing.

The counsellor provides crisis therapy to patients and primarily supports their relatives. Their responsibilities also include coordination and maintaining contact with the Swedish Social Insurance Agency, insurance companies, the Swedish Public Employment Service, and employers.

Assessments of activities are conducted by an occupational therapist (OT). The OT focuses on teaching compensatory strategies and, when necessary, testing assistive devices. Activity- or occupation-based interventions are used to enhance participation. The OT evaluates routines, hobbies, home environment activities, and work tasks, providing advice on how to modify activities to make them easier for patients to perform.

The medical secretary manages administration and is available by telephone during office hours. They can quickly contact other OBIT team professionals and, if required, arrange a consultation at the earliest opportunity.

During treatment, patients have regular hospital visits, telephone calls, or telemedicine consultations with at least 1 OBIT professional. Initially, following enrolment or if the patient’s psychological status worsens, OBIT may offer weekly or biweekly contact, though most of the time contacts are less frequent. The frequency of contact and treatment is carefully tailored to the patient’s needs.

Patients are also offered group sessions where they meet others in similar situations, and lectures are provided by the various OBIT professionals. Education, both in groups and individually, is an important part of the programme.

The treatment is individualized but follows a structured programme. All patients have rehabilitation plans, which are updated every 3–6 months. Common goals in the rehabilitation plan include achieving a better balance between activity and rest in daily life through energy-saving strategies and learning memory techniques, with the overall aim of managing everyday life and work as effectively as possible. It is important for patients to understand the normal reactions and emotions commonly experienced, such as fear, anger, anxiety, irritation, sadness, and hopelessness.

When patients have learned to compensate for their disabilities and achieved as stable a life situation as possible, OBIT treatment is concluded through a structured process. Typically, patients are referred to their general practitioner. In a few cases, treatment is discontinued due to lack of motivation to continue rehabilitation. Patients must not have active substance abuse during treatment.

The mean treatment period for all patients discharged from OBIT since 1998, including those excluded from this study, was 2 years and 6½ months (range: 1 month to 8 years).

This study started in March 2021, as soon as the study was approved by the Swedish Ethical Review Authority (ref no 2020-07044 0315).

### Controls

**Fig. 1:**
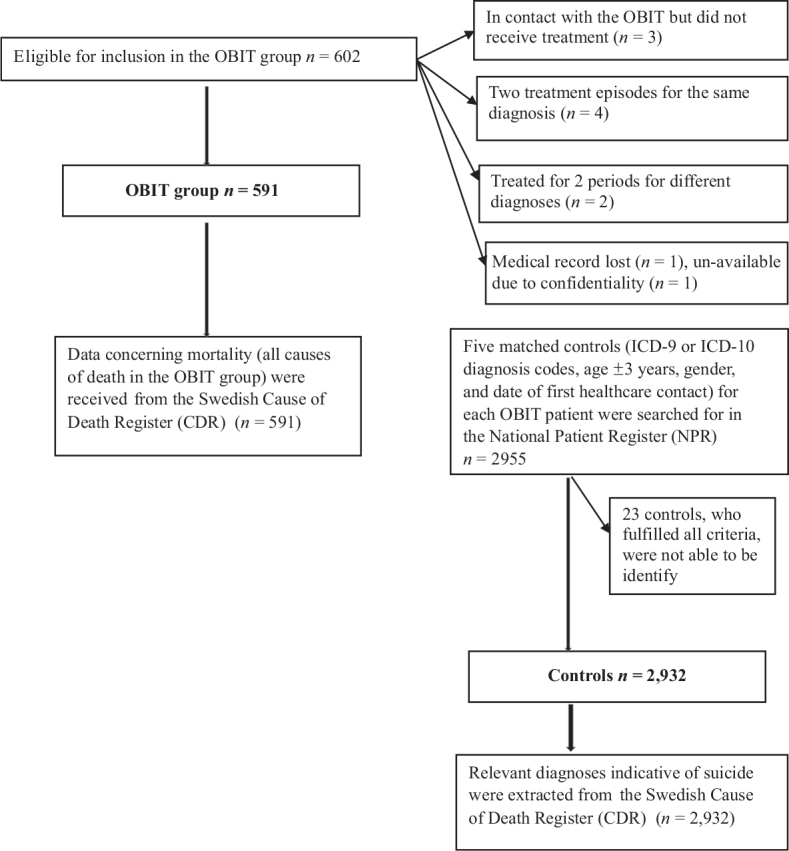
Study flowchart.

Data were obtained from the Swedish Cause of Death Register (CDR), and 5 controls for each OBIT patient were identified in the National Patient Register (NPR) maintained by the National Board of Health and Welfare. For the OBIT group, data on all causes of death were requested from the CDR, while for the controls, relevant diagnoses indicative of suicide were extracted. Controls were matched by ICD-9 or ICD-10 diagnosis codes, age ±3 years, gender, and date of first healthcare contact ±5 years (see flowchart, Fig. 1).

### Statistical analysis

The statistical analyses were conducted as follows. Tests of differences between proportions were performed using Fisher’s exact test. Proportions of patients are presented as numbers and percentages, and differences between proportions are presented with 95% confidence intervals (CI). Where applicable, relative risks with 95% CI are also provided. To enable statistical analysis of the relative risk, it was necessary to assume one suicide in the OBIT group for the sensitivity analysis. All analyses were two-sided with an alpha of 0.05, and calculations were performed using SAS 9.4 (SAS Institute, Cary, NC, USA).

## RESULTS

A total of 602 patients were eligible for inclusion in the OBIT group. Of these, 3 patients were assessed but did not receive treatment. Four patients had 2 treatment episodes for the same diagnosis, and 2 patients had been treated for 2 periods for different diagnoses; only the first treatment period was included. One medical record was lost, and 1 was unavailable due to confidentiality. This resulted in a final OBIT group of 591 patients.

It was not possible to identify 5 controls meeting all matching criteria for 7 OBIT patients. For 1 patient with the ICD-10 diagnosis I678 (Other specified cerebrovascular disease), 1 control was missing. For 1 patient with the ICD-10 diagnosis B941 (Sequelae of viral encephalitis), 1 control was missing, and for 2 additional patients with the same diagnosis, 4 controls each were not found. For 1 patient with the ICD-9 diagnosis 139W (Late effects of other infectious and parasitic diseases corresponding to ICD-10, B94.9), 4 controls were missing, as were 4 controls for 1 patient with the ICD-10 diagnosis G04A (Other encephalitis, myelitis and encephalomyelitis). For another patient with the ICD-10 diagnosis G04A, no controls were found. The control group therefore comprises 2,932 individuals, with a total of 23 controls not found (0.007 %) as shown in Fig. 1.

A post-check conducted by the statistician confirmed that the controls were adequately matched for age and gender. The diagnoses (ICD-10), gender and age at onset of illness in the treated group and the controls are presented in Table I.

**Table I:** Diagnoses (ICD-10), gender, and age at onset of illness in the treated group (OBIT group *n* = 591) and the controls (*n* = 2,932)

Diagnoses (ICD-10)	OBIT *n* = 591 *n*	OBIT%	Controls *n* = 2,932 *n*	Controls %
Stroke (I60, I61, I62, I63, I67, I69)	272	46.0	1359	46.3
Concussion (S060)	147	24.9	735	25.0
TBI, other (S061–S09)	107	18.1	535	18.2
Infectious diseases (CNS) (A87, B02, B94, G03, G04, G05, G06, B94.9)	22	3.7	92	3.1
Anoxic brain damage (G93)	18	3.0	90	3.0
Benign neoplasms (D32, D33, D35, D43)	13	2.2	65	2.2
Malignant neoplasms (C71)	6	1.0	30	1.0
Malformation of cerebral vessels (Q28.2 Q 28.3)	4	0.07	20	0.07
Epilepsy (G40)	1	0.02	5	0.02
Other degenerative diseases (G31)	1	0.02	5	0.02
Gender				
Male	315	53.3	1563	53.3
Female	276	46.7	1369	47.7
Age at onset of illness				
Mean	42.3 (12.8)		42.6 (13.2)	
Median	45 (12; 63)		45 (9; 66)	

For categorical variables, % (*n* = ) is presented.For continuous variables, mean (SD)/median (min; max) are presented.

For the OBIT-treated group data on all causes of death were obtained from the CDR.

Fifty-nine patients in the OBIT group had died by December 2020. The causes of death are presented in Table II at the chapter or ICD-10 block level, referring to the ICD-10 block code ranges as organized in ICD-10.

**Table II:** Cause of death in the OBIT group to December 2020 (*n* = 59)

Variable	ICD-10	Primary diagnosis (*n* = 59) *n*
Cause of death		
Diseases of the circulatory system	I100-I99	24
Neoplasms	C00-D48	11
Diseases of the respiratory system	J100-J99	9
Certain infectious and parasitic diseases	A00-B99	4
Cerebrovascular infarction	I63	3
Cerebrovascular bleeding	I61	3
Diseases of the digestive system	K00-K93	2
Endocrine, nutritional and metabolic diseases	E00-E90	1
Sequel of external causes	V01-Y98	2*
Gender	*n*	
Male	47	
Female	12	
Age at death		
Mean (SD)/median (min; max) are presented.	64.4 (9.6) 65 (51; 78)	

* One death was caused by a transport accident and 1 by poisoning by drugs, medicaments, or biological substances.

None of the deceased patients had suicide recorded as the cause of death.

None of the deceased patients in the OBIT group had suicide recorded as the cause of death. One patient died from intoxication. A review of their medical records revealed no information suggesting suicide.

For the control group, we obtained information from the CDR on whether the controls had any cause of death indicating suicide. The diagnoses requested were:

- ICD-10: Intentional self-harm X60–X84, Event of undetermined intent Y10–Y34, and Unspecified effects of external causes (including intentional self-harm suicide) T76.
- ICD-9: Suicide and self-inflicted injury E950–E959, Homicide and injury purposely inflicted by other persons 9600–9899, and Injury undetermined whether accidentally or purposely inflicted E980–E989.

We also requested data from the CDR on the occurrence of the following causes of death, as they could indicate possible suicide: drowning T751, toxic effect of carbon monoxide T58, toxic effect of hallucinogens or other drugs including antidepressants T40, T43, T50, alcohol T51, and hypothermia T68.

Forty individuals in the control group were found to have diagnosis codes associated with suicide; see Table III.

**Table III:** Diagnoses (ICD-10) associated with suicide among controls

Cause of death	ICD-9 and ICD-10	Verified and probable (*n* = 40) *n*	Verified (*n* = 26) *n*
Event of undetermined intent	Y10-Y34	14	
Intentional self-harm	X60-84	25	25
Self-inflicted injury	E9571	1	1

Fourteen of these 40 patients had one of the following diagnoses: event of undetermined intent Y10–Y34, which were interpreted as probable suicides.

Twenty-six had the ICD-10 diagnosis of intentional self-harm (X60–X84; *n* = 25) or ICD-9: suicide and self-inflicted injury E9571 (*n* = 1), which were interpreted as verified suicides.

No patients in the control group had any of the following diagnoses: homicide and injury purposely inflicted by other persons (9600–9899), injury undetermined whether accidentally or purposely inflicted (E980–E989), or unspecified effects of external causes including intentional self-harm (T76).

There were 20 additional patients in the control group with cause of death diagnoses relevant to this study: drowning T751 (*n* = 4), toxic effect of carbon monoxide T58 (*n* = 3), toxic effect of hallucinogens or other drugs T40, T43, or T50 (*n* = 9), alcohol T51 (*n* = 3), and hypothermia T68 (*n* = 1). Some of these cases may represent suicides, but this could not be verified, and they were therefore not included in the statistical calculations.

Table IV lists diagnoses associated with the brain injury, gender, and age at death among the controls with probable and verified suicide.

**Table IV:** Diagnoses associated with the brain injury, gender, and age at death of controls with verified and probable suicide (*n* = 40) and among the controls with verified suicide (*n* = 26)

Variable	Controls (*n* = 40) % (*n*)	Controls (*n* = 26) % (*n*)
Brain injury		
TBI	55.0 (22)	53.8 (14)
Stroke	22.5 (9)	26.9 (7)
Anoxic brain injury	12.5 (5)	15.4 (4)
Encephalitis	2.5 (1)	3.8 (1)
Tumour	7.5 (3)	0.0 (0)
Gender		
Male	75.0 (30)	76.9 (20)
Female	25.0 (10)	23.1 (6)
Age at death	44.5 (11.7)46.5 (22; 71)	46.6 (11.4)47 (22; 71)

For categorical variables, % (*n* = ) is presented.For continuous variables, mean (SD)/median (min; max) are presented.

The diagnoses associated with the brain injury among the deceased individuals did not differ considerably between the groups of 40 or 26, except for the frequency of tumour.

All tumour cases had diagnoses coded as Y10–Y34 (event of undetermined intent).

### Statistical results

Two statistical calculations were performed: 1 including all 40 probable and verified suicides and 1 including only the 26 verified suicides.

There were 0 (0.0%) primary endpoints – suicide, including probable suicides – in the OBIT group and 40 (1.4%) in the control group. The difference in percentage was 1.4 (95% CI 0.9 to 1.8, *p* = 0.001). Assuming 1 (0.2%) suicide in the OBIT group, the relative risk (RR) was 0.12 (95% CI 0.02 to 0.90). Assuming 8 suicides in the OBIT group would yield an event rate similar to that of the control group.

Calculating for 26 (0.9%) verified suicides in the control group, the difference in percentage was 0.9 (95% CI 0.6 to 1.2, *p* = 0.015). Assuming 1 (0.2%) suicide in the OBIT group, the relative risk (RR) was 0.19 (95% CI 0.03 to 1.40). Assuming 5 suicides in the OBIT group would yield an event rate similar to that of the control group.

### Data from the reviews of the medical records (n = 102)

Medical records from all OBIT team members were reviewed for a randomly selected subgroup of the OBIT group (*n* = 102). The selection was limited to records from May 2007 onwards, when the hospital’s medical records became fully computerized, ensuring complete data. The number of reviews was also constrained by limited research funding.

All 102 patients had received OBIT interventions for at least 6 months. These patients had been discharged from further treatment by the OBIT during 2011–2020.

The aim was to describe the OBIT group according to risk factors for suicide and the targeted treatment. One aim was therefore to describe the occurrence of psychiatric deceases, suicide attempts, and substance/alcohol abuse before the brain injury and to examine and describe the frequency of depression and the treatments for depression that these patients had received.

There were a total of 21 patients among the 102 participants who had a history of substance misuse and/or psychiatric diseases. Of those, 17 had documented psychiatric diseases. One of the 17 had documented psychosis, and 2 had been diagnosed with bipolar disorder. Ten had documented alcohol misuse, 3 in combination with narcotic misuse. Three patients had made suicide attempts before the injury.

During treatment by the OBIT, there was no tolerance for substance abuse.

An assessment for depression had been conducted during OBIT treatment for all 102 patients. Of these, 70 patients (69%) were estimated to have experienced depression at least once during treatment, and of those, 10 had documented suicidal ideation. Three patients were referred for psychiatric consultation.

One patient had neither contact with a psychologist nor received antidepressant medication. The remaining 69 patients received the following interventions for depression through OBIT: 42 patients received both antidepressant medication (SSRI/SNRI) and contact with a psychologist, 18 patients had contact with a psychologist only, and 9 patients received antidepressant medication only.

Forty patients were prescribed sleeping pills. Of the 32 patients among the 102 who were not depressed, 5 were prescribed sleeping pills for at least 1 period during treatment.

## DISCUSSION

As no patients in the OBIT group died by suicide during treatment or follow-up until 2020, compared with 26, or possibly as many as 40, in the control group, we estimate that OBIT treatment may have prevented up to 8 or more suicides.

The OBIT has reported that no patients died by suicide during treatment between 2021 and December 2025.

The review of medical records supports the observation that most OBIT patients experience depression following their illness or injury, at least for a period during the first years and sometimes later during the rehabilitation phase, as also described by Teasdale and Engberg (1, 24) and Eriksson et al. (2).

Studies have examined and described interventions to prevent depression and suicide after stroke, TBI, or other brain injuries. Joubert et al. (25) presented a 2008 prospective randomized study on an “integrated care” (IC) model, in which the primary care physician and stroke specialist regularly communicated and reported to each other. Follow-up occurred every 3 months with repeated depression assessments for 1 year. The IC group had significantly lower depression scores at the 12-month follow-up, leading the authors to conclude that the integrated care approach provides a framework for detecting and monitoring depressive symptoms and appears protective against post-stroke depression. Simpson and colleagues reported in 2007 positive results from a project where health and disability services providers had suicide prevention training for people with TBI (26) and another paper about the important role of general practice in the long-term support of people with TBI (27). Silverberg and Panenka (14) reported in 2019 on depression after TBI, noting that at least 1 in 5 individuals with TBI, regardless of injury severity, meet the criteria for a Major Depressive Episode within the first 6 months. They state that SSRIs are the first-line treatment among antidepressants and conclude that health providers should routinely screen and initiate treatment for depression after TBI. However, the evidence for antidepressant efficacy following TBI has been mixed and inconclusive (28).

Roy et al. (29) emphasize the importance of psychotherapeutic interventions to address the individual’s perception of social impairment in the early TBI period. The approaches described in these studies align closely with OBIT treatment, which provides regular, long-term follow-up and aims to detect and treat depression as early and effectively as possible. Accessibility to professionals is also considered crucial.

Furthermore, the absence of suicide in the OBIT group may be attributed not only to preventive treatment but also to the professional management of severe depression. The team approach is important, as all members observe and report early signs of depression, which can develop at any stage during rehabilitation. The physician promptly prescribes antidepressants and provides guidance on their use, while the psychologist can begin treatment without delay. All team members provide support and encouragement, offering patients hope for the future.

Most patients with depression are adequately treated by the team; however, if there are alarming signs, such as severe suicidal ideation, patients are referred to a psychiatric clinic for consultation. In rare cases, the police may be involved in transporting a patient to the hospital, although this is very uncommon.

Other aspects of OBIT treatment may also contribute to the positive outcomes. Understanding that symptoms and functional impairments result from the injury rather than a psychiatric illness is important for patients’ crisis management. Training in managing fatigue and activity limitations can reduce frustration in daily life and at work. Rehabilitation for brain injury takes time to yield positive results.

Group treatment is valuable, as patients can realize they are not alone and share experiences on using compensatory strategies. Sleep disturbances and stress reactions are common after brain injury; education on sleep mechanisms and pharmacological treatment can reduce fatigue and frustration, while teaching stress management, relaxation, and mindfulness techniques can indirectly help prevent depression.

Involving relatives is also important, as they can provide support and alert the team if the patient’s behaviour changes or they express more negative thoughts.

Another important factor is that the treatment is individualized according to each patient’s needs and the time required to adapt to their new life, which can vary considerably between individuals. The timing of the different interventions is also crucial.

The mean age at death in the suicide group of 40 was 46.5 years (range 22–71). In the OBIT group (non-suicide-related deaths), the mean age at death was 64.25 years (range 51–78), which is unsurprising given that most deaths were due to medical conditions (see Table III). Complete CDR data were not requested for the control group.

More men than women died by suicide in the control groups, with similar proportions in both the group of 40 probable and verified suicides and the group of 26 verified suicides. Male sex has been identified as a risk factor in a large Swedish cohort study (*n* = 7,140,589) by Crump et al. (30). This contrasts with studies by Stenager et al. (31) and Teasdale and Engdahl (1) in Denmark, as well as Forsström et al. (32) in Finland, which found female sex to be a risk factor in stroke populations. The differing diagnoses in these studies, compared with our cohort with a variety of brain injury aetiologies, may explain this discrepancy.

This study has several strengths and limitations. One strength is the inclusion of a control group, which was well matched for diagnosis, age, gender, and timing of care. The number of dropouts was small.

Another strength in this study is that a review of the medical records shows that patients with pre-injury substance misuse and psychiatric illnesses have been accepted for treatment and thus are included in the OBIT group. It is well known that these pre-injury factors elevate suicide risk after a brain injury (24), but nevertheless there are no suicides in the OBIT group. This supports our hypothesis that the treatment is effective in preventing suicide and that it is also protective for high-risk patients. A limitation is that we do not have any data concerning these pre- and post-injury factors for the controls.

The researchers and authors are familiar with the programme, having been involved with it for many years, which may be both a strength and a limitation. While the programme is straightforward to describe, there is a risk that the authors may overinterpret the results. Another limitation is that the exact number of suicides in the control group cannot be determined, as there are some uncertainties regarding the registered causes of death. In a study by Tøllefsen et al. (33), 88% of deaths recorded as suicide in the Swedish dataset were confirmed, and a further 21% of undetermined deaths were reclassified as suicide. Overall, the level of certainty for reported suicides was 77% in the Swedish dataset, with the greatest uncertainty for poisoning-related deaths. In our view, the figure of 26 suicides is unlikely to be overestimated.

Medical records were reviewed for a subgroup of the OBIT group. We consider, however, that the 102 patients are representative for describing the frequency of depression and its treatment.

There was an unusually long delay before we could begin cooperation and contacts with the National Board of Health and Welfare, as all register studies on COVID-19 were given the highest priority at that time. The study started in 2021, and the results were received in 2023 and 2024. It was conducted in 2 phases because obtaining data from the registers was more expensive than anticipated, requiring additional funding, which explains the extended timeline. We do not believe, however, that this has affected the results.

In conclusion, this study supports that low-intensity, long-term treatment by a specialized outpatient rehabilitation team is effective in preventing suicide during treatment and in the years following discharge. The patients have received sustained, multidisciplinary support over several years, which likely contributed to their adjustment following a life-altering injury. Most patients experienced depression during rehabilitation and received appropriate treatment. We suggest that a programme combining strategies for managing a changed life situation and treatment of depression is crucial for positive outcomes and should be made available to more patients with brain injury.
